# Platelet function and Isoprostane biology. Should Isoprostanes be the newest member of the Orphan-ligand family?

**DOI:** 10.1186/1423-0127-17-24

**Published:** 2010-04-06

**Authors:** Harold J Ting, Fadi T Khasawneh

**Affiliations:** 1 Department of Pharmaceutical Sciences, College of Pharmacy, Western University of Health Sciences, Pomona, California 91766, USA

## Abstract

While there have been many reports investigating the biological activity and signaling mechanisms of isoprostanes, their role in biology, particularly in platelets, appears to still be underestimated. Moreover, whether these lipids have their own receptors is still debated, despite multiple reports that discrete receptors for isporpstanes do exist on platelets, vascular tissues, amongst others. This paper provides a review of the important literature of isoprostanes and provides reasoning that isoprostanes should be classified as orphan ligands until their receptor(s) is/are identified.

## Review

Maintaining proper function of platelets is vital as their primary task is to stop bleeding from an injured vessel, a process known as hemostasis [1,2]. The hemostatic plug that forms in order to halt blood loss must be capable of rapid dissolution upon wound healing [3]. Nonetheless, blood flow must remain unimpeded in all other instances to ensure effective nutrient and waste exchange. Thus, platelets are, necessarily, firmly regulated blood elements that must be highly and quickly responsive to activating stimuli but otherwise are "completely" quiescent. Malfunctions in either of these behaviors leads to a host of disorders [3,4]. Furthermore, various deficiencies in activation result in bleeding diseases which are associated with morbidity and mortality and may require lifetime treatment (e.g., von Willebrand disease) [4,5]. Conversely, improper activation, or recruitment of platelets to sites where hemostasis is not needed are hallmarks of myocardial infarction, ischemic stroke, peripheral artery disease and other thrombotic ailments that together represent a major source of mortality [6]. Thus, the mechanism of platelet regulation and more specifically, their activation is of great interest as understanding these signaling pathways will allow for the development of specific and rationally developed therapeutic intervention strategies.

Platelets are the second most abundant cells of the blood numbering hundreds of millions per milliliter of whole blood [7]. Yet, this still only comprises a very small fraction of blood volume, as they are individually minuscule. This derives from the fact that platelets are not themselves "true" cells but are merely cellular fragments [8]. Thus, they lack nuclei; which makes certain modifications to their signaling or effector molecules irreversible (e.g. nonspecific cyclooxygenase inhibition when platelets are exposed to aspirin) [9]. Platelet function returns only upon replacement with newly synthesized cells. To this end, platelets are produced in the bone marrow and are derived from very large cells called megakaryocytes [10]. As megakaryocytes develop, they undergo a budding process that results in the release of several thousand platelets per megakaryocyte allowing for rapid replenishment in the absence of faults in platelet regulation [8,10].

## Platelet Activation

While a platelet lacks several organelles that are present in other cell systems, it possesses complex structures that are essential for its central role in hemostasis; which can be inappropriately marshaled in thrombosis-based events. Platelets are normally smooth and discoid in shape, hence their name [11]. If platelets are stimulated by one of a group of agonists (thrombin, thromboxane A_2 _(TXA_2_), ADP, etc) they initiate and undergo a sequence of physiological and anatomical changes [1,11-15]. The first discernible sign of platelet activation is shape change (i.e., platelets become spherical), and is associated with the extension of long pseudopodia [16]. This is due to an elevation in actin and myosin to levels that are only exceeded by muscle cells and is initiated by increases in cytosolic calcium (Ca^2+^) that results in phosphorylation of myosin light chain by a Ca^2+^-calmodulin-dependent kinase, which in turn enhances myosin binding of actin [1,17]. In fact, experimentally induced activation can be achieved through exposure to Ca^2+ ^ionophores in addition to physiological agonists and/or their derivatives [18].

Platelets also express adhesive proteins on their surface that allows them to adhere to the exposed subendothelium in a injured blood vessel, as well as to surface proteins of nearby platelets [2,11]. Therefore, the next phase of activation is characterized by adhesion and aggregation of platelets as they bind to the damaged tissue as well as each other, thereby preventing further blood loss from a wound. In addition, platelets contain several types of intercellular granules (i.e., alpha and dense granules) [19]. Alpha granules contain growth factors (such as platelet-derived growth factor, insulin-like growth factor-1, tissue growth factor-β, and platelet factor-4), the adhesion molecule, P-selectin, and clotting proteins (such as thrombospondin, fibronectin, and von Willebrand factor) [20]. Dense granules contain platelet agonists such as adenine nucleotides (ADP), ionized Ca^2+^, and signaling molecules (such as histamine, serotonin, and epinephrine) [21,22]. Secretion is considered the next stage of platelet activation, as these chemicals play an essential role in the hemostatic process as they serve to amplify platelet response [13]. Due to this exponential activation, many of these steps overlap among a population of platelets. Hence, aggregation is reinforced by the secreted fibrinogen and thrombospondin, further binding the platelets together, as well as by the dense granule-secreted agonists which can signal further secretion (thus providing a strong positive feedback loop). These substances are thought to potentiate each others' effects. Finally, actin and myosin mediate platelet retraction as activated platelets condense the loose clot formed previously to seal a vascular wound into a hard, dense mass capable of resisting dispersion until wound healing is complete [23].

## Platelet Signaling

Central to platelet activation is the mobilization of Ca^2+ ^from stores within the platelet that then signals additional Ca^2+ ^entry into the cell from the extracellular environment. In this connection, the Ca^2+ ^ionophore A23187 mediates platelet shape change, aggregation, and secretion, essentially acting identically to other platelet agonists [18]. The particular temporal arrangement of platelet activation is believed to be a result of increasing concentrations of Ca^2+ ^and possibly other intracellular signaling transmitters. The responses appear to be chronological, but this is not due to any prerequisites of a previous stage but because of the order of their dependence on Ca^2+ ^concentration [1,24]. Thus, since shape change requires the least Ca^2+ ^concentrations to trigger, it's the most difficult to inhibit. On the other hand, secretion and aggregation require greater Ca^2+ ^concentrations, and, consequently, are more readily inhibited. The signaling pathways controlling the initiation or the amplification of intracellular Ca^2+ ^entry are thus of major interest in platelet biology. While there are a host of additional effectors, comprised of G-proteins, MAP Kinases, and other molecules, these all integrate at the level of activating the GPIIb/IIIa on platelet surface [25]. When platelets are activated, this adhesive molecule undergoes a conformational change so that it can recognize fibrinogen molecules, which allows for the formation of platelet aggregates [16,25].

**Figure 1:**
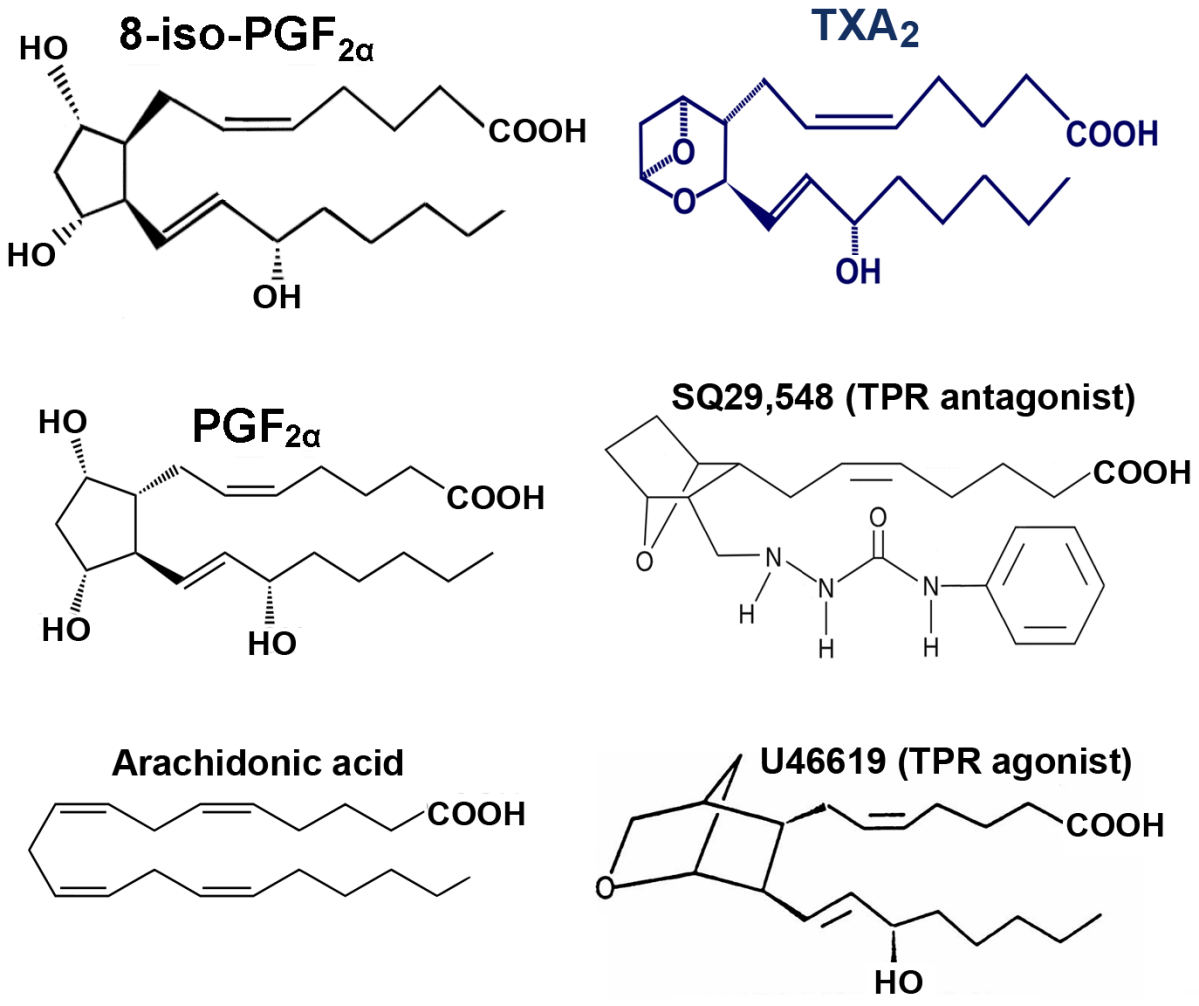
**Structure of arachidonic acid (the precursor for all prostaglandins), various TPR ligands, PGF_2α_, and the most abundant isoprostane 8-iso-PGF_2α_**.

**Figure 2:**
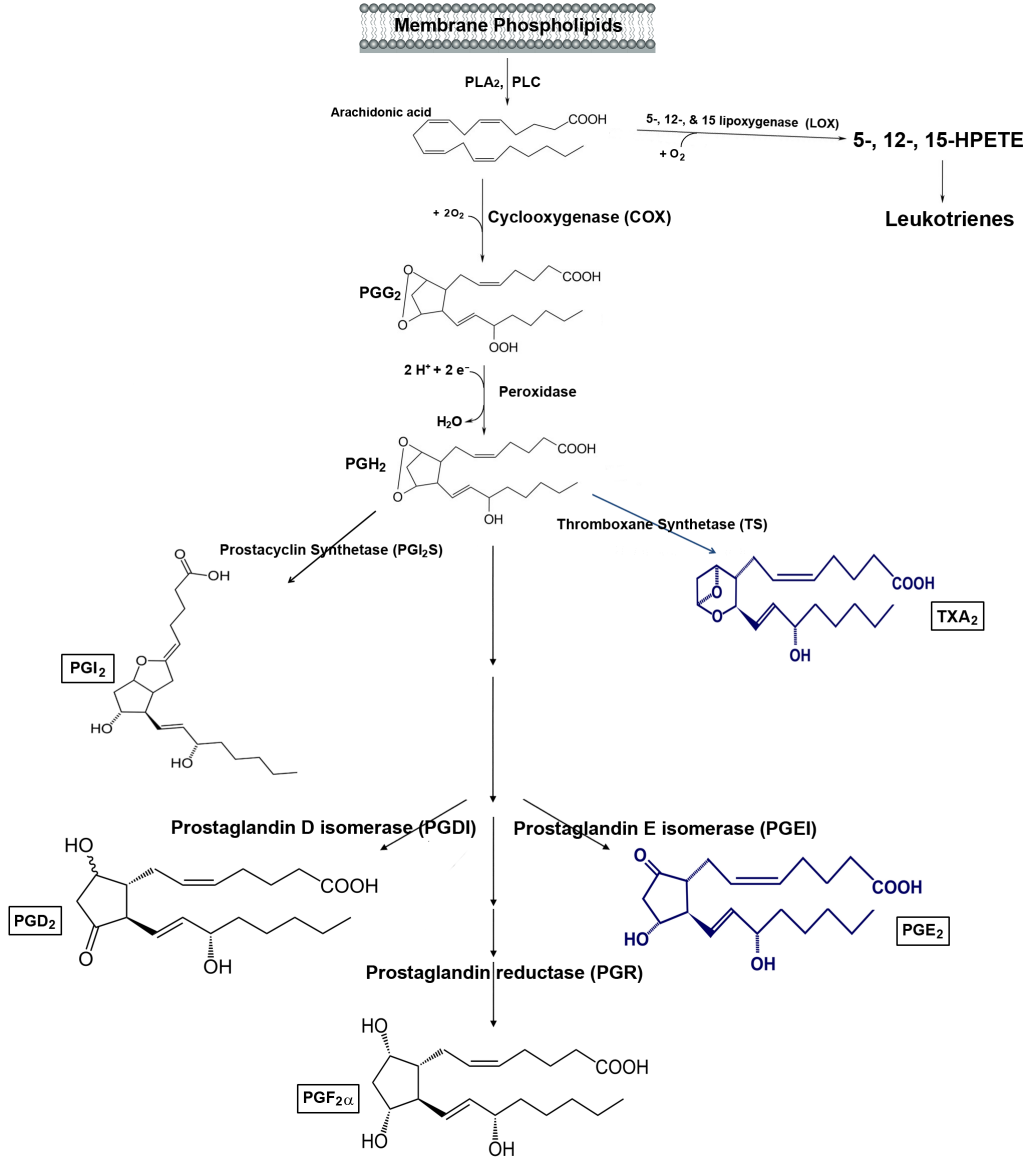
**A schematic representation of the arachidonic acid metabolism pathway**. After its liberation by phospholipases, ((i.e., phospholipase A_2 _(PLA_2_) or phospholipase C (PLC)), the free arachidonic acid may undergo enzymatic metabolism by the lipoxygenases which produce HPETEs and leukotrienes, and the cyclooxygenases (COX-1, COX-2) which generate prostaglandins and thromboxanes. The specific repertoire of the arachidonic acid metabolites produced may vary according to the expression profile of these enzymes in different cell types. In platelets, for example, arachidonic acid is metabolized by COX-1 into the prostaglandin endoperoxides, PGG_2 _and PGH_2_. Next, thromboxane synthetase further metabolizes PGH_2 _into TXA_2_, which is a potent activator of platelet aggregation, with a half-life of 20-30 seconds. Thromboxane A_2 _is then hydrolyzed to the inactive form TXB_2 _(not shown). On the other hand, if PGH_2 _is metabolized by prostacyclin synthetase, then PGI_2 _would be produced (e.g., in endothelial cells). Furthermore, if PGH_2 _is acted upon by PGD or PGE isomerase, then PGD_2_, and PGE_2 _are produced, respectively (e.g., in renal cells). Finally, if the PG reductase metabolizes PGH_2_, then PGF_2α _is produced (e.g., pulmonary vessels). Thus, the biological functions of arachidonic acid are exerted indirectly after its metabolism into prostaglandin and thromboxane metabolites.

Platelets are activated through several signaling modalities. Aggregation initiates within seconds upon exposure to ADP, thrombin, serotonin, and epinephrine. Thrombin is considered the most potent physiologic agonist and thus has been widely used to study secretion along with arachidonic acid (AA), endoperoxides, or TXA_2 _(Figure 1) as they can induces platelet shape change, aggregation, and secretion [26]. In contrast, platelet stimulation by epinephrine is not associated with change in platelet shape [27]. Additionally, the effects of "low" concentration of collagen are thought to be dependent on arachidonate metabolism. Aggregation is usually required for secretion as the dense packing and resultant decrease in interstitial spaces serves to concentrate otherwise low levels of released AA metabolites [13,28]. One exception to this requirement is thrombin as it can induce secretion in nonaggregated suspensions [1]. Due to the presence of numerous, biologically active metabolites, one critical activation arm of platelets is dependent on AA. AA, which is the most abundant, is a 20-carbon unsaturated fatty acid [29]. The release of AA from the membrane by phospholipases, and subsequent metabolic modifications leads to the formation of well-characterized prostaglandins and thromboxanes (Figure 2). Of primary importance to platelet function is the formation of TXA_2_, which is generated from arachidonic acid in reaction catalyzed by the platelet cyclooxygenase-1 enzyme [30]. Generated TXA_2 _then binds to its G-protein coupled receptor (GPCR) known as TXA_2 _receptor (abbreviated as TPR). There are two splice variants for TPR with distinct tissue expression, i.e., the placental α-isoform and the endothelial β-isoform [31]. Interestingly, using isoform-specific TPR antibodies, TPR-α but not TPR-β was immunoprecipitated from platelets [32]. Furthermore, consistent with this finding, platelets were found to express high levels of mRNA for the α-isoform and low levels of β-isoforms. Taken together, these data suggest a limited role, if any, for the β-isoforms in platelet function.

Interaction of TXA_2_, or other agonists to their cognate receptors, leads to transduction of activating signals into secondary messengers. One major pathway for this response is the GPCRs [29,33-35]. G-proteins, which consist of three different subunits, α, β and γ, can be divided into four major families, G_q_, G_12_, G_i _and G_s_, of which platelets have been found to express several distinct members [34,36]. More specifically, a host of *in vitro *approaches involving reconstitution studies, affinity copurification experiments or cross-linking studies with photoactivated GTP analogs demonstrated that platelets express G_q_, G_16 _(G_q _family), G_12_, G_13 _(G_12 _family), G_s_, as well as G_o_, G_i _and G_z _(G_i _family) [33,35,37-41]. These studies have specifically revealed that TPR couples to the G_q _and G_13 _isoforms. Additionally, U46619, a stable TXA_2 _mimetic, induces a rapid, transient rise in intracellular Ca^2+ ^in platelets and in HEK293 cells cotransfected with G_αq _or G_α11 _and the α-isoform of TPR [42]. Further evidence also indicates that the TPRα isoform can functionally couple to G_q _or to G_11 _*in vivo*.

The G-protein, Gα_q_, signaling pathway starts by the activation of phospholipase C (PLC) which in turn metabolizes phosphatidylinositol 4,5-bisphosphate (PIP_2_) into inositol 1,4,5-trisphosphate (IP_3_) and diacylglycerol (DAG) [43,44]. IP_3 _then binds to its receptor and raises cytosolic Ca^2+ ^concentrations by inducing Ca^2+ ^release from vesicles into the cytoplasm [45,46]. DAG serves to stimulate protein kinase C (PKC) which in turn activates phospholipase A_2 _(PLA_2_) [47]. It is thought that both the increase in cytoplasmic Ca^2+ ^and the production of DAG are necessary for full platelet activation, and lead to the activation of the glycoprotein GPIIb/IIIa[48,49]. This GP is a heterodimeric complex of two GPs on the platelet surface that serves as the fibrinogen receptor [16,25]. Fibrinogen is a dimeric molecule that serves as a molecular bridge which crosslinks platelets, thereby enabling platelet aggregation and formation of a primary hemostatic plug [50]. On this basis, activation of GPIIb/IIIa is absolutely critical for platelet function. Under *in vitro *settings, the conformational change required for the formation of "active" GPIIb/IIIa requires calcium [48,49,51]. Taken together, it's believed that increases in intracellular Ca^2+ ^are the ultimate mediator of activation in platelets.

Arachidonic acid metabolites such as TXA_2_, have been shown to trigger platelet responses dependent on stimulation of G_12/13_-/G_q_-coupled receptors [37,38,41,52]. Signaling through these receptors has been shown to enhance phosphorylation of several tyrosine kinase families (Src, Syk and FAK) [53]. Consistent with the role of G_12/13_-coupled receptors, low doses of U46619 was found to trigger tyrosine phosphorylation of FAK, Syk and Src [54]. Secretion of TXA_2 _(or other AA metabolites that act though TPRs such as isoprostanes) from activated platelets and other sources may then mediate further activation through this tyrosine-kinase-dependent signaling pathway [55]. Additionally, thrombin has been reported to induce phosphorylation of FAK in both platelets and HEK293 cells, and binding of GPIIb/IIIa to fibrinogen initiates a second sustained wave of tyrosine phosphorylation [56,57]. In fact, GPCR-mediated activation of tyrosine kinases is well characterized during integrin-mediated assembly of cytoskeletal and signaling proteins to focal adhesion sites [58]. Interestingly, U46619 mediated activation was found to be independent of GPIIb/IIIa binding to fibrinogen or the interaction of secreted ADP with its platelet receptors (i.e., P2Y_1 _and/or P2Y_12_) [54]. Signaling through this modality alone was insufficient to stimulate full platelet activation, but synergized with the G_z_-linked adrenaline receptor (epinephrine) to mediate platelet aggregation [29,59,60]. In fact, it has been reported that combined signaling via G_12/13 _and G_i _is required for full platelet activation [61,62]. Furthermore, signaling through both the G_12/13_-dependent Rho-kinase, and the tyrosine-kinase-dependent pathways was found to be required for the synergistic activation of GPIIb/IIIa [63]. Thus, these signals converge with additional signals ensuing from the engagement of G_z_-coupled receptors [33,36]. Together, this data reveals that a combination of agonists at subthreshold levels or with low potency can serve to activate platelets in the absence of more potent and perhaps more intentional activation.

Collectively, platelet TPRs are known to couple to the four major families of G-proteins, which in turn activate numerous downstream effectors, including second messenger systems such as IP_3_/DAG, cAMP, small G proteins (Ras, Rho, and Rac, effectors such as p160 ROCK, as well as the Ca^2+^/calmodulin system) [33,34,36,64-67], phosphoinositide-3(PI3) kinase, activation of Syk, Src, and FAK tyrosine kinase and mitogen-activated protein kinase (MAPK, specifically p38 and p42) as well as protein kinase A and C (PKA and PKC) [54,65,68]. Additionally, the action of many platelet agonists (ADP, thrombin, low dose collagen) serves to mediate synthesis and subsequent secretion of TXA_2 _[1,49,63]. Thus, TXA_2 _is not only a potent direct activator of platelet function, it is also a key effector in other agonist mediated pathways. Fortunately, TXA_2 _is also highly unstable (a half life of around 30 seconds) and functions primarily as an autocrine or local paracrine signal allowing for tight spatial regulation of platelet activation [69]. The discovery of this central role for AA metabolite pharmacological activity has motivated the design of drugs with TPR antagonistic activity.

## Isoprostanes

**Figure 3:**
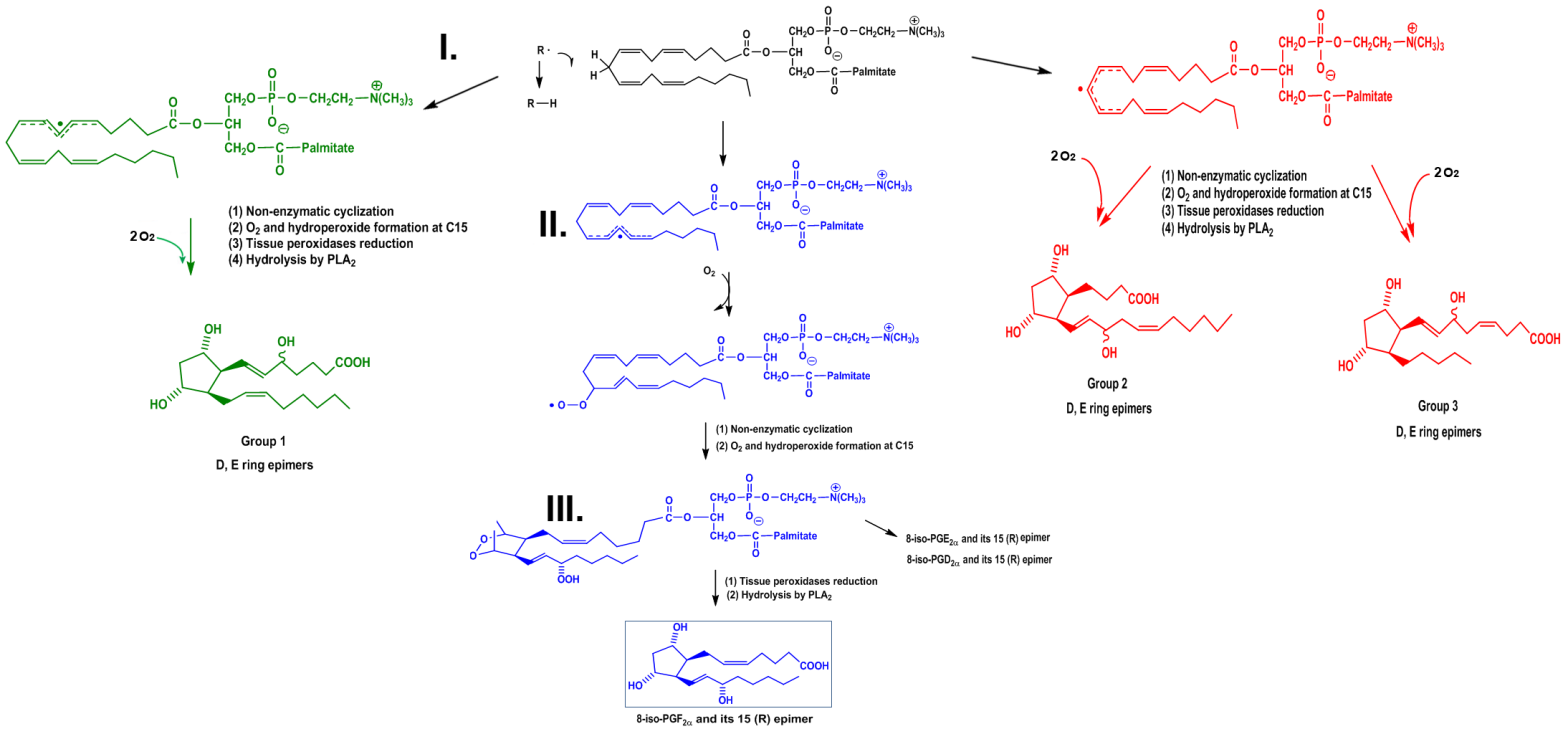
**A schematic representation of the metabolic cascade for the non-enzymatic generation of isoprostanes**. This is a proposed scheme in which four series of regioisomers of PGG_2 _are formed, before they are reduced to PGF_2α _isomers. As shown, isoprostanes can be formed from arachidonic acid in situ in phospholipids, from which they are presumably cleaved by phospholipases A_2_. PGG_2 _spontaneously rearranges to PGD_2 _and PGE_2 _thereby generating isoprostanes of the D and E series. The initial step in the formation of an isoprostane from arachidonic acid **(I) **is the generation of a lipid free radical by the abstraction of a hydrogen atom from one of the three methylene-interrupted carbon atoms, C7, C10, or C13, as shown here, by a free radical (FR•) which may be a hydroxyl radical (HO•), a superoxide radical (O_2_^-^•) or other free radical, and results in **(II)**. Radical attack at C-10 is shown, abstraction at the other positions determines the relative proportion of the isomers formed. The lipid free radical is converted to a peroxy radical by reaction with molecular oxygen. The peroxy radical cyclizes in an intramolecular reaction that yields an endoperoxide **(III)**. The free radical chain reaction will continue to propagate until quenched by an antioxidant.

While research on arachidonic acid metabolites have focused on the traditional enzyme mediated pathway, there is another potential route for arachidonic acid modification, i.e., a free radical mediated pathway [70,71]. This metabolic cascade has led to the investigation of a class of "naturally" occurring prostaglandin-like products known as isoprostanes. These are produced by the free radical mediated oxidation of unsaturated fatty acids (Figure 3) in membrane phospholipids as opposed to the enzymatically catalyzed oxidation found with the classical AA derivatives such as TXA_2 _[70,72]. As the formation of isoprostanes is not enzymatically-directed, but random chemical degradation, there is a larger variety of molecules produced *in vivo *(Figure 3). Whereas the endoperoxide prostaglandin G_2 _(PGG_2_) is specifically formed by the cyclooxygenase enzymes (COX-1 and COX-2), four classes of isoprostanes are produced as a result of the free-radical oxidation of AA (Figure 3), with each class containing 16 subtypes of isoprostanes resulting in 64 individual isoprostane molecules [73].

Due to their interesting chemical properties and large number of distinct members, isoprostanes are of clinical interest for two main reasons: 1. they are ligands for prostaglandin receptors, and thus may exhibit biological activity like TXA_2 _and other AA metabolites [70,74]; and 2. they have been found to associate with the oxidative status of an organism [75,76]. Moreover, there is evidence that their levels serve as a predictor of the onset and severity of inflammatory diseases such as atherosclerosis and Alzheimer's disease [75,77]. Indeed, isoprostanes are thought to participate in the pathogenesis of Alzheimer's disease. Evaluation of the blood and urinary levels of certain isoprostanes' and their metabolites, respectively, has been demonstrated to be a reliable approach to the assessment of lipid peroxidation, and therefore of oxidative stress *in vivo *[78]. More specifically, evidence points to the possibility that isoprostanes may be involved in the genesis of certain disease states. For example, *in vitro *studies revealed that isoprostanes can induce oligodendrocyte progenitor cell death and induce vasoconstriction and mitogenesis, as well as inflame endothelial cells to bind monocytes, a critical initiating event in atherogenesis [79-81]. An *in vivo *mouse model suggested that isoprostanes are involved in the development of thrombi at sites of vascular injury [82]. Furthermore, LDLR- and ApoE-deficient mouse models demonstrated that these oxidation products accelerate the development of atherosclerosis independent of *de novo *TXA_2 _synthesis or changes in plasma lipid levels [83]. In patients with atherosclerosis and acute myocardial infarction, levels of isoprostanes were also found to be elevated and their reduction coincided with decreased atherogenesis, suggesting a role for this oxidized lipid in the development of this disease state [76,84].

Most of the studies examining the biological activity of isoprostanes have been conducted with a specific form, 8-iso-PGF_2α _(Figure 1), as it is one of the most abundantly produced *in vivo *[85]. Much work has been done with this compound as it is commercially available, having been previously synthesized for unrelated reasons and was therefore readily available for a host of studies (i.e., infusion, bioassay, receptor binding/affinity studies, etc). Additionally, it exhibits chemical stability that significantly exceeds that of TXA_2_, suggesting it's potential for long-term signaling capacity that may lead to systemic priming of platelets [83]. To this end, 8-iso-PGF_2α _has been reported to exhibit significant biological activity. Specifically, it has been found to be a mitogen in 3T3 cells and in vascular smooth muscle cells and evidence suggests it may play a role in pulmonary oxygen toxicity [86,87]. This biological activity may be a result of modification of the integrity and fluidity of membranes, a characteristic consequence of oxidative damage [88]. This occurs as a result of the distorted shape of isoprostanes relative to the normal fatty acids present in membrane phospholipids and could be critical in modifying the hemodynamic properties in vascular tissues into a more dysfunctional microenvironment conducive to initiating chronic disease states.

## Isoprostane Signaling Pathways

Given the plethora of reports that suggest 8-iso-PGF_2α _exerts biological actions on platelets, elucidating the concentrations necessary to elicit these effects and reconciling these with the levels reported to circulate *in vivo *is of relevance to investigating its underlying mechanism of action. In pursuit of this goal, it was found that there is a minimum threshold concentration of 8-iso-PGF_2α _at which it has the capacity to induce platelet shape change and above which it can alter the formation of thromboxane or irreversible aggregation in response to platelet agonists [89,90]. Additionally, 8-iso-PGF_2α _synergistically mediates aggregation upon exposure to subthreshold concentrations of platelet agonists [74]. Such a modality is supported by findings that when epinephrine and AA were added to platelet rich plasma (PRP) in subthreshold concentrations, they acted in a synergistic manner to produce platelet aggregation[29]. This synergistic platelet activation in response to dual exposure to 8-iso-PGF_2α _and other agonists would be most likely in settings where platelet activation and enhanced free radical formation (and thus isoprostane formation) coincide, a characteristic microenvironment of atherosclerosis. This synergism was found to be abrogated by calcium channel inhibitors, an α_2_-receptor antagonist and inhibitors of PLC, MAP kinase, and COX pathways [29]. Since increased cytosolic Ca^2+ ^is essential to platelet activation, the proposed mechanism for potentiation between platelet agonists is the activation of the Ca^2+ ^signaling cascade. Thus, a rise in cytosolic Ca^2+ ^levels induced by the first agonist primes platelets for an enhanced functional response to a second agonist. In accord with this possible mechanism, increasing concentrations of 8-iso-PGF_2α _resulted in dose-dependent, irreversible platelet aggregation in the presence of subthreshold concentrations of collagen, ADP, AA, and analogs of TXA_2 _(i.e., I-BOP, U46619)[74]. This phenomenon was not evident when platelets were pretreated with either COX inhibitors or TPR antagonists, indicating a clear dependence of aggregation on the secondary formation of TXA_2_. Interestingly, 8-iso PGF_2α _failed to desensitize the calcium or inositol phosphate responses to platelet stimulation by these agonists. Furthermore, 8-iso-PGF_3α _a related chemical to 8-iso-PGF_2α _failed to initiate platelet shape change or aggregation nor did it raise intracellular calcium or inositol phosphates, suggesting a structural requirement for engaging the receptor's ligand binding domain(s).

In the course of characterizing the properties of isoprostanes, it was discovered that they exert their biological activity on a host of cell types: platelets, kidney, and others, presumably via the activation of TPR [80,91,92]. It has previously been shown that 8-iso-PGF_2α _induces intracellular Ca^2+ ^mobilization in cells co-transfected with TPR_α _and G_αq _or G_α11 _[42]. More specifically, co-transfection of G_α11 _produced greater mobilization of intracellular Ca^2+ ^than that stimulated by G_αq_. Surprisingly, in human platelets, 8-iso PGF_2α _failed to cause a dose-dependent increase in TPR_α _phosphorylation, in spite of stimulating inositol phosphate formation [32]. It is possible that the capacity of 8-iso-PGF_2α _for *in vivo *platelet activation manifests only if it's delivered through an especially concentrated mechanism, such as from microvesicles shed by activated cells, or through selective reincorporation of secreted isoprostanes into the membrane[93]. Nevertheless, this explanation is only partially satisfactory since the TXA_2 _mimetic U46619, but not 8-iso-PGF_2α_, reduced glomerular insulin space and increased inositol 1,4,5-trisphosphate production in rat glomeruli and mesangial cells in a an apparently TPR-dependent fashion (i.e., blocked by the TPR antagonist SQ29,548)[91]. Conversely, rat aortic smooth muscle cells were found to possess specific binding sites for both TXA_2 _and 8-iso-PGF_2α _and displayed functional responses to both agonists, such as time- and dose-dependent activation of MAP kinases [74,91]. Interestingly, the addition of 8-iso-PGF_2α _and U46619 together did not potentiate or antagonize the maximal level of Ca^2+ ^mobilized in either platelets or transfected HEK293 cells, which suggests that 8-iso-PGF_2α _and U46619 are acting through the same pathway (TPR) [42]. In line with this notion, SQ29,548 was found to be equally potent in abolishing the Ca^2+ ^response in both platelets and transfected HEK293 cells upon stimulation with either U46619 or 8-iso-PGF_2α_. Pretreatment of platelets or transfected cells with thrombin, on the other hand, did not desensitize the rise in intracellular Ca^2+ ^upon subsequent stimulation with either U46619 or 8-iso-PGF_2α_, which provides further evidence that these lipids share a common signaling pathway, though previous work showing abrogation of effect by 8-iso-PGF_2α _in the presence of COX inhibitors suggests that formation of TXA_2 _is the potential link at the TPR modality [74].

Studies have also revealed that 8-iso-PGF_2α _stimulates platelet shape change and reversible aggregation through a TPR-mediated process [74]. In support of this, 8-iso-PGF_2α _was found to be a potent vasoconstrictor in the rat lung and kidney, which was specific through TPRs[81,92]. Furthermore, a TPR antagonist was shown to block 8-iso-PGF_2α_-induced vasoconstriction of renal glomeruli, carotid arteries, and vascular smooth muscle cells [92,94,95]. Additionally, it was found that the proatherogenic effect of 8-iso-PGF_2α _is mediated via TPR activation and is secondary to the induction of specific inflammatory mediators, such as sICAM-1 and MCP-1 but not ET-1, at the site of lesion development [83]. On the other hand, several reports disputed the notion that the stimulatory effects of 8-iso-PGF_2α _are primarily mediated through TPRs, adding more complexity to this issue. The primary alternative signaling mechanism predicts the existence of unidentified discrete isoprostane receptors in human platelets and smooth muscle cells, the basis for which is found in studies detailing differences between the potencies of 8-iso-PGF_2α _and TPR agonists in inducing DNA synthesis and MAP-kinase activation [74,83,91,96,97]. Further complicating matters, this alternative proposal has also been recently disputed with several possible explanations for the noted discrepancies such as variations in the experimental conditions/cellular preparations, or inherent differences in the potency of the ligands employed [94]. In summary, there are clear ambiguities concerning the mechanisms by which isoprostanes modulate cellular function.

**Figure 4:**
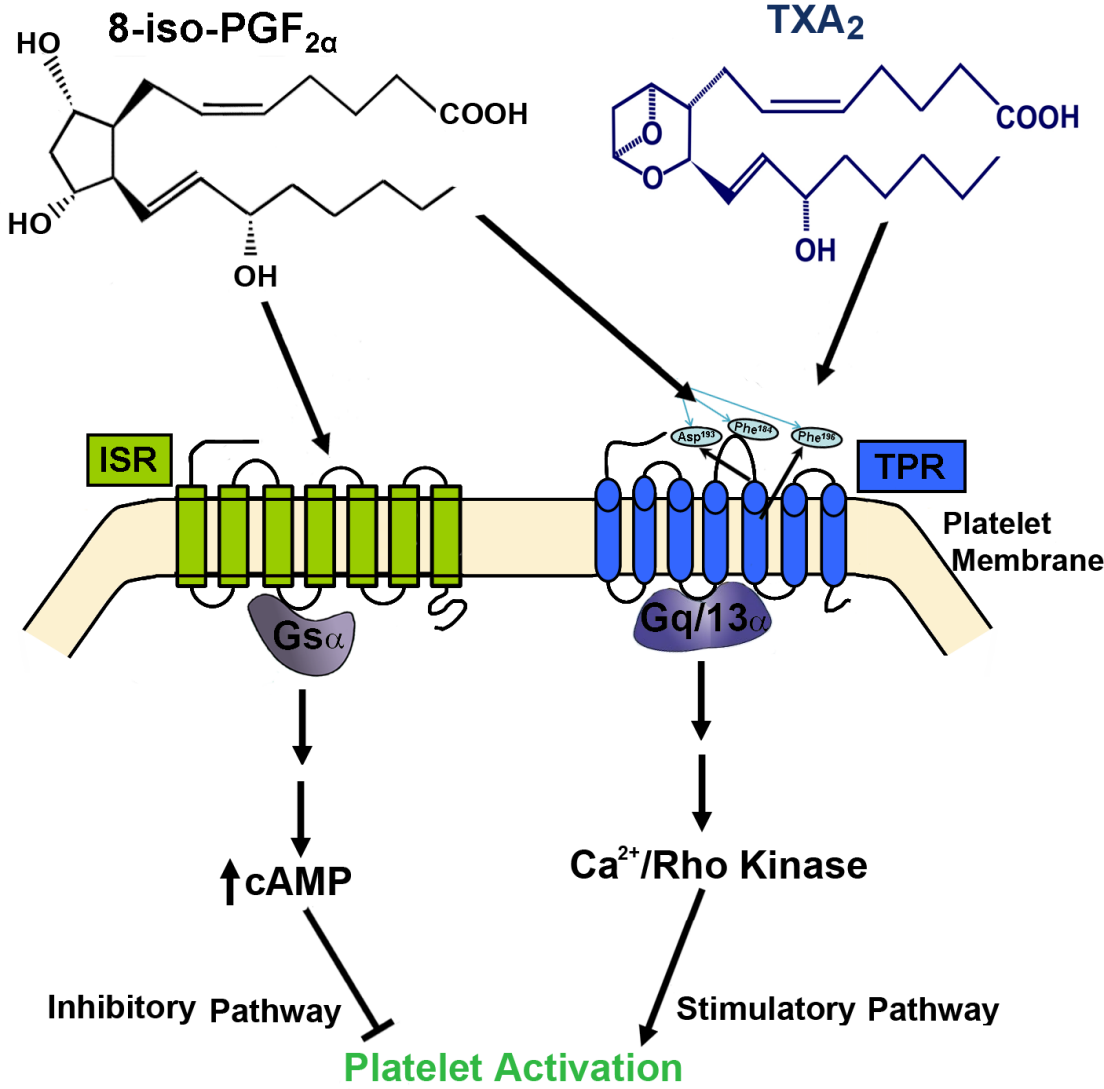
**Schematic representation of a model describing the inhibitory and stimulatory signaling pathways for TPR-dependent modulation of platelet activation by 8-iso-PGF_2α_**.

As a distinct and further confounding layer of complexity it has been recently reported that 8-iso-PGF_2α _signals through both stimulatory and inhibitory pathways in platelets and that this inhibition by 8-iso-PGF_2α _operates through a cAMP-dependent mechanism (Figure 4) [70]. Additionally, reduction of isoprostane formation by vitamin E in combination with the suppression of TXB_2 _biosynthesis (a metabolic marker of TXA_2_) was shown to be more effective than the two approaches alone in experimental atherosclerosis [98]. In this connection, by blocking TXA_2 _synthesis, aspirin (ASA) appears to facilitate increased isoprostane production from AA, which in turn, may amplify the anti-thrombotic effects of ASA itself through a secondary inhibitory process. Taken together, it might be predicted that a therapeutic regimen combining ASA along with a TPR antagonist would be more beneficial than therapy with ASA alone. Specifically, under these conditions, the isoprostane stimulatory effects would be blocked by TPR antagonism, while its inhibitory effects would be promoted by elevating the levels of circulating isoprostane. Thus, specific isoprostane-receptor interactions may mediate agonist activation of one effector pathway, yet act as an antagonist for an alternate pathway.

## Alternative Isoprostane Signaling Pathways

Despite this body of evidence associating elevated isoprostane with oxidative stress and vascular disease pathology, as well as supporting a potential role for isoprostanes in mediating a host of disease processes such as apoptosis, brain cell damage, and thrombosis, their biological activity and signaling mechanisms remain poorly understood. A major hindrance to teasing out the mechanism(s) is that specific inhibition of isoprostanes is not universally reported. Aside from prostaglandin H_2_-TXA_2 _and isoprostanes, the TPR receptors share other endogenous ligands such as HETE. Moreover, other AA derivatives (free radical-dependent or otherwise) may be biologically relevant and signal through TPR, thus further obfuscating the activity of isoprostanes on platelet biology [99]. One of the most promising avenues for research is thus isolating the contributions of signaling through the TPR which is known to competently bind to isoprostanes. Studies report ligation of both existing membrane and nuclear prostaglandin receptors by isoprostanes [100,101]. However, the possibility of signaling through other isoprostane receptors is raised by studies reporting an apparent inability of isoprostanes to ligate or signal efficiently through either TPR isoform *in vitro*, despite evidence that their *in vivo *actions are mediated by TPR [91,94].

One potential alternative signaling mechanism posits a contribution by the phenomenon of GPCR heterodimerization, which is a result of a specific receptor having multiple isoforms, or non-isoform receptors that can freely dimerize with each other. Heterodimerization has been reported to alter receptor properties such as regulation and ligand binding affinity [102]. In addition, studies indicate that GPCR heterodimers may mediate changes in the signaling preferences/characteristics of the individual receptors [100,102-104]. An example is found in the dimerization of the β1 and β2 adrenergic receptors, which enhances cAMP formation in response to isoproternol and has also been implicated in regulating cardiac contractility [105]. Similarly, dimerization of the alpha and beta isoforms of the TPR has been shown to mediate alterations in both receptor regulation and signaling [103,104]. Consistent with previous reports, 8-iso-PGF_2α _stimulated TPR-mediated IP_3 _generation less potently than IBOP and U46619 in cells expressing TPR_α _or TPR_β _individually. In contrast, while cells stably expressing both TPR_α _and TPR_β_, exhibited significantly enhanced IP_3 _generation following treatment with 8-iso-PGF_2α_, this was not the case with IBOP or U46619. This finding was not due to preferential binding to an isoform or in combination as there were no differences in the capacity for 8-iso-PGF_2α _to displace the TPR antagonist SQ29,548 in membranes generated from TPR_α_, TPR_β _or TPR_α_/TPR_β _co-expressing HEK cells despite signaling more efficiently through a TPR_α_/TPR_β _heterodimer. However, it has been reported that SQ29,548 does not fully occupy the binding site for 8-iso-PGF_2α _in the TPR_α_/TPR_β _heterodimer. These data together indicate that heterodimerization does not modify the well characterized TPR binding site, but instead may create an alternative isoprostane binding site. Additionally, the possibility exists that downstream G protein coupling is modified with GPCR heterodimerization. For example, if the TPR_α_/TPR_β _heterodimer were more efficiently coupled to Gq in co-transfected cells it might be expected that IP_3 _and calcium signals would be elevated. However, the absence of a similarly enhanced signaling response with IBOP or U46619 stands in contradiction to this hypothesis. Finally, it's difficult to infer/interpret the biological relevance of the impact of TPR_α_/TPR_β _heterodimer formation on isoprostane biology in platelets given that platelets do not express TPRTPR_β_.

Yet another potential mechanism for isoprostane mediated signaling is found at signal transduction, whereby the response following activation of GPCR's is altered; this is a particularly enticing avenue for future investigation since chronic disease states such as atherosclerosis are characterized by persistent, subacute levels of dysregulation. In this connection, following their activation, dissociated Gα subunits may not bind to their originally coupled GPCR receptors. Instead, the final equilibrium of the reassociation process for liberated Gα is determined by the relative expression and affinity of the various activated GPCR's[106]. To illustrate, following PAR1 receptor activation, both the level of PAR1 presentation and its Gα affinity would decrease as PAR1 is internalized following activation along with receptor alterations due to PAR1/ligand interactions. Together, these effects would promote increased Gα coupling to TPRs and thus a consequent shift to a higher ligand affinity state for this receptor. Expression/affinity-mediated TPR/G-protein coupling raises the possibility of competition for G-proteins between TPRs and other GPCRs, and helping to define the predominant signaling pathways through which TPRs signal under different experimental conditions and in different cell types. In support of this hypothesis, it was found that activation of Gα_i_-coupled receptors increased the potency and the efficacy of inositol phosphate production induced by bradykinin or UTP activation [106]. In addition, other studies demonstrated synergistic interactions between U46619 and ADP as well as U46619 and epinephrine [59,60,107,108].

## Isoprostane Binding

Due to these sometimes confounding reports on isoprostane signaling, attempts have been made to elucidate the specific segment(s) that define the receptor ligand-binding pocket of isoprostanes to TPR's, which will also address the question of whether isoprostanes can physically interact with TPRs or not. We, recently reported that 8-iso-PGF_2α _coordinates with specific residues on platelet TPR's and that Phe^196 ^(Figure 4) specifically serves as a unique TPR binding site for this ligand [70]. Furthermore, it was revealed that TPRs exhibit ligand specificity, in both G-protein and TPR cotransfected HEK293 cells as well as in platelets. Consistent with previous reports regarding the relative potency, the maximal Ca^2+ ^response observed in platelets was 3- to 4-fold greater after stimulation with U46619 than with 8-iso-PGF_2α _[42]. This is critical as the signaling in platelet activation appears to integrate at the level of elevating intracellular Ca^2+^. Previously it was noted that 8-iso-PGF_2α _signals through both stimulatory and inhibitory pathways in platelets and that the inhibitory effects of 8-iso-PGF_2α _operated through a cAMP dependent mechanism (Figure 4). This is supported by reports that 8-iso-PGF_2α _interacts with platelets at two separate binding sites [70,74,91]. One of these sites was found to mediate a small rise in intracellular Ca^2+^, a concomitant increase in inositol phosphates and protein kinase C activation as well as supporting irreversible platelet aggregation, when stimulated by TXA_2_/PGH_2 _analogs. The other site mediates the majority of the calcium released from intracellular stores and platelet shape change [109,110]. Additionally, as mentioned elsewhere, the rapid, agonist-induced phosphorylation of TPR_α _appears to involve signaling through low affinity binding sites. This was verified in studies using platelets pretreated with GR32191 (which blocks the low affinity TPR sites) where it was found that neither low concentrations of I-BOP, nor high concentrations of agonist resulted in TPR_β _phosphorylation[109].

## Isoprostane *in vivo *Levels

In discussing isoprostanes it is important to note that isoprostanes can be produced *in vivo *at levels several orders of magnitude higher than classical prostaglandins/thromboxanes, and that they remain largely stable in circulation in comparison to ligands such as TXA_2 _itself [69,71]. Consequently, the biological effects of these signaling modalities could, in theory, have a substantial systemic impact on cellular functions along a broad temporal range, characteristic of chronic disease states. Furthermore, it is known that the *in vivo *levels of isoprostanes can be enhanced by the presence of vascular disease, thus further associating this oxidative marker to the chronic dysfunction characterized by oxidative stress [76,77,84]. However, one obfuscating complication remains in deducing the role of isoprostanes in mediating platelet activation; this derives in part from the fact that the reported EC_50 _concentrations of isoprostanes required to elicit functional responses in platelets are much higher than their measured concentrations in the circulation, even in syndromes of oxidant stress [74]. The highest plasma levels recorded in patients remain outside the range of concentration necessary to evoke biological responses in platelets or in other cell types. Thus, 8-iso-PGF_2α _does not likely function as a conventional, circulating hormone *in vivo*, and even potential autocoidal functions may necessitate highly concentrated forms of delivery to local receptors. Nonetheless, it's possible that these lipids do achieve such concentrations locally (compartmentalization), and hence modulate platelet function at punctuate microenvironenments conducive for their effect. Another possible explanation to this potential conflict is that incidental activation of TPR receptors by 8-iso PGF_2α _may contribute at subthreshold levels to the adverse effects of oxidant stress *in vivo *as would be the case with some of the alternative signaling modalities described previously.

## Conclusion

An alternative to the classical COX-mediated AA modification pathway has more recently been identified, that of chemical degradation. More specifically, free radical-induced oxidative modification of AA, which results in the production of a group of chemicals called isoprostanes [71,81]. Furthermore, isoprostanes can circulate *in vivo *at concentrations orders of magnitude higher than other AA metabolites such as TXA_2 _and remain much more chemically stable (Table 1) [111-115]. This family of lipid-mediators, particularly 8-iso-PGF_2α_, has been strongly correlated with the oxidative microenvironments found in various disease states. Many reports suggest that isoprostanes produce their biological activity by directly interacting with TPRs (e.g., on platelets), and a plethora of reports indicate they are associated with increased risk of several vascular diseases. This association manifests in a broad range of cell types but almost all appeared dependent on mediating TPR activation, and secondarily, several G-proteins. Further complicating the task of elucidating its underlying mechanism of effect, reports have revealed that 8-iso-PGF_2α _signals through both stimulatory and inhibitory pathways in platelets. While the identity of the receptor that mediates its inhibitory effects remains unknown, evidence indicates that it's coupled to Gs. And this is indicative of the continued need for further research in this field as there are often conflicting reports on the activity and signaling pathways of this class of chemicals; possibly due to the subtle nature of their contribution to platelet activation. Taken together, this suggests the possibility that in chronic and sustained dysregulated states as found in vascular disease, isoprostanes could possess a significant systemic impact on cellular functions without initiating an acute thrombotic event in the absence of other agonists and as such remains an intriguing area of further research.

**Table 1:** A comparison between certain biological properties of TXA_2 _and 8-iso-PGF_2α_

Lipid	Half life **(T**_1/2_**)**	Plasma Concentration (endogenous)	Method of synthesis	Receptors
**TXA**_2_	20-30 seconds^111^	TXB_2 _(1-66 pg/ml)^113^	Enzymatic^26^	TPR_α _& TPR_β_^31^

**8-iso-PGF**_2α_	1-10 minutes^112^	351-1831 pg/ml (dinordihydro metabolite)^114^	Non-emzymatic & enzymatic^73,115^	TPR_α_^80 ^& TPR_β_^74,91 ^and ISR^70,74,97^

## Abbreviations

TXA_2_: thromboxane A_2_; TPR: thromboxane A_2 _receptor; AA: arachidonic acid; GPCR: G-protein coupled receptor; Ca^2+^: calcium

## Competing interests

The authors declare that they have no competing interests.

## Authors' contributions

FTK: Prepared the manuscript and figures; HJT: Manuscript preparation, and reference formatting.
